# Regret-sensitive treatment decisions

**DOI:** 10.1186/s13561-018-0198-2

**Published:** 2018-08-06

**Authors:** Yoichiro Fujii, Yusuke Osaki

**Affiliations:** 1 Faculty of Economics, Osaka Sangyo University, Nakagaito 3-1-1, Daito-shi, Osaka, 574-8530 Japan; 2 Faculty of Commerce, Waseda University, Nishi-waseda 1-6-1, Shinjuku-ku, Tokyo, 169-8050 Japan

**Keywords:** Medical decision-making, Non-expected utility, Public medical service, Regret and rejoicing, Treatment threshold, D81, I12

## Abstract

The threshold approach to medical decision-making, in which treatment decisions are made based on whether the probability of sickness exceeds a predetermined threshold, was introduced by (Pauker and Kassirer, N Engl J Med 293:229-234, 1975) and (Pauker and Kassirer, N Engl J Med 302:1109-1116, 1980). This study generalizes the threshold approach using regret theory. Regret theory is one of the established alternatives to expected utility theory (EUT), and partly overcomes the descriptive limitations of EUT. Under regret theory, agents suffer disutility from regret or enjoy utility from rejoicing by comparing the chosen alternative with the forgone one. We examine the effect of regret and rejoicing on the threshold approach by setting the EU case as a benchmark, and show conditions under which regret and rejoicing monotonically change the threshold probability. The threshold probability is lowered by regret and rejoicing under the reasonable condition in the sense that the condition can explain observed choices that EU fails to describe. This suggests that agents opt to undergo medical treatment by the feeling of regret and rejoicing. This result might explain the social problems that occur in relation to the public provision of medical services in many OECD countries such as medical expenditure rising faster than government forecasts. The results also imply that regret sensitivity might cause inequality of benefits from public medical services. Finally, we offer a solution to this problem.

## Background

In their semsinal research, Pauker and Kassirer [1, 2] introduced an important normative criterion into medical decision-making under risk named the “treatment threshold.”

Under the treatment threshold approach, medical decision-making is related to the probability of sickness at which agents are indifferent between treatment and no treatment. This probability is referred to as the “threshold probability.” If the probability of sickness is higher (lower) than the threshold probability, agents should (should not) undergo medical treatment. Since Pauker and Kassirer’s work, a number of studies have examined applications of the treatment threshold.^1^ However, most of these studies are based on expected utility theory (EUT), which has been the dominant tool in medical decision-making under risk.

Empirical observations of decision-making under risk often contradict EUT (e.g., Allais [3] and Kahneman and Tversky [4]), and many preference representations have been proposed to explain these observations. Bell [5] and Loomes and Sugden [6] incorporated both regret and rejoicing into preference representations in what they termed “regret theory.” In regret theory, agents not only gain utility from the chosen alternatives, but also suffer disutility from regret or enjoy utility from rejoicing by comparing the chosen alternative with the forgone one.^2^ Regret theory can be viewed as one of the “bold” alternatives to EUT.^3^ That is, regret theory has accumulated numerous empirical studies, developed axiomatic foundations for preference representation, and been applied to various economic analyses.^4^

Somewhat surprisingly, regret theory has received scant attention in the field of medical decision-making. Thus, the present study bridges the gap between the importance of regret theory and its lack of application to medical decision-making. Agents often face difficulties in medical decision-making because their decisions can have a serious impact on their lives. This suggests that the forgone alternatives play an essential role in medical decision-making in addition to the chosen alternatives. Regret theory can formally incorporate the effect of the forgone alternatives into medical decision-making. It enables us to examine how regret and rejoicing influence the threshold probability in the abovementioned treatment threshold approach. By introducing a weight for regret and rejoicing sensitivity, we show the condition under which the threshold probability is decreasing in the weight. In other words, more regret-sensitive agents tend to opt for treatment at lower sickness probabilities. This condition gives us the shape of the function that captures preferences for regret and rejoicing, which is called the “regret–rejoicing function.” This shape seems to be reasonable based on the classical paper by Bell [5] because it succeeds in explaining the observed choices, which are inconsistent with EUT.

Regret theory has already been introduced into the treatment threshold approach by Djulbegovic et al. [7] and Hozo and Djulbegovic [8].^5^ However, there are two significant differences between our study and earlier studies. The first is that earlier studies assumed the regret function to be linear, whereas this study considers the regret–rejoicing function to be nonlinear. Second, previous studies introduced the concept of “acceptable regret”^6^ and examined how regret affects the threshold probability. In contrast, this study determines the threshold probability and examines how regret sensitivity affects it. Thus, this study succeeds in overcoming the problems encountered by earlier studies.^7^ The first difference is essential because preferences toward regret and rejoicing can be captured by the shape of the regret–rejoicing function, and a linear regret–rejoicing function can identify EUT.^8^ The shape of the regret–rejoicing function is crucial for treatment decisions, and we could obtain the wrong implications from regret theory if the shape was incorrect.

This result has implications in relation to issues surrounding the public provision of medical services. Many OECD countries collect taxes and/or public insurance premiums to provide universal health-care programs (UHCPs). However, this is a controversial issue from both political and public viewpoints. Thus, regret sensitivity might help to explain the following problems related to UHCPs: government spending on medical services is much higher than forecast, and only a small minority of the population enjoys the benefits of UHCPs. We also suggest a solution to this problem.

The rest of this paper is organized as follows. In “Methods” section, we introduce preference representation, obtain the threshold probability, and determine the effect of regret on such a probability. “Results” section presents some of the implications of the results regarding the social problems that occur in the public provision of medical services in OECD countries. We also suggest a solution to these problems based on our analysis. “Discussion” section concludes.

## Methods

### Regret-sensitive preference representation

Here, we describe regret theory, as formulated by Bell [5] and Loomes and Sugden [6]. An agent has two alternatives: one is chosen and the other is forgone. *w*_*c*_ and *w*_*f*_ denote the consequences of the chosen alternative and the forgone alternative, respectively. In addition, to derive the utility from the actual outcome of the chosen alternative, the agent feels *regret* if the forgone consequence is preferable to the chosen one and *rejoicing* otherwise. We adopt the specific utility form that appears in Theorem 1 of Bell [5]. The utility function of the regret theory has the following form:

1 $$ U\left({w}_c\right)+k\cdotp g\left(U\left({w}_c\right)-U\left({w}_f\right)\right) $$

We call (1) the regret-sensitive utility function and consider an agent to be regret-sensitive if his/her preference is represented by the utility form (1).^9^ Here, *U* is a risk function that is assumed to be continuous and increasing, while *g* is a regret–rejoicing function that is also assumed to be continuous and increasing. Note that neither convexity nor concavity for both the risk function *U* and the regret–rejoicing function *g* is necessary for the analysis.^10^

Next, we consider the case in which the consequence of the forgone alternative is preferable to that of the chosen alternative, that is, *w*_*c*_ < *w*_*f*_. In this case, an agent suffers disutility *k* · *g*(*U*(*w*_*c*_) − *U*(*w*_*f*_)) < 0 by regret for his/her choice compared with a better forgone alternative. In the opposite case, that is, *w*_*c*_ > *w*_*f*_, an agent gains utility *k* · *g*(*U*(*w*_*c*_) − *U*(*w*_*f*_)) > 0 by rejoicing about his/her choice compared with a worse forgone alternative. A constant, *k* ∈ [0, +∞), expresses how an agent places a weight on the regret–rejoicing term. We sometimes call the agent “agent *k*” when the weight is *k* with all other things being identical. The strength of regret sensitivity can be compared using *k*. A formal definition is as follows.

**Definition 1.** *Let us consider two agents*, $\overline{k}$ *and* $\underline{k}$*. Agent* $\overline{k}$ *is more regret-sensitive than agent* $\underline{k}$ *if* $\overline{k}$ *is more than* $\underline{k}$*.*

Because agents have the same risk function and regret–rejoicing function, the difference in regret sensitivity among agents can be completely captured by *k*. We note that the utility function corresponds to EUT for *k* = 0^11^, and the original formulation by Bell [2] and Loomes and Sugden [6] for the case that *k* is normalized to unity.

### Treatment decision

We incorporate regret sensitivity into the classical treatment decision under diagnostic risk by Pauker and Kassirer [1, 2]. Let us consider an agent who develops particular symptoms and has a regret-sensitive utility function of (1).^12^ Following Pauker and Kassirer [1, 2], we consider the following setting. There are two health states: the agent is either sick (*s*) or healthy (*h*). In addition, there are two treatment alternatives: treatment (*T*) or no treatment (*NT*). The agent needs to choose either *T* or *NT* before knowing his/her true health state. Quality-adjusted life years (QALYs) are determined by the health state and treatment choice.^13^ The agent gains utility from the realized QALYs and feels either regret or rejoicing based on the difference in utility between the realized QALYs and the forgone QALYs.

Let ${Q}_h^T\ \left({Q}_s^T\right)$ denote the agent’s QALYs when he/she undergoes treatment and is healthy (sick). Similarly, ${Q}_h^{NT}\ \left({Q}_s^{NT}\right)$ denotes the agent’s QALYs when he/she does not undergo treatment and is healthy (sick). We assume that ${Q}_h^{NT}>{Q}_h^T>{Q}_s^T>{Q}_s^{NT}$, and set $U\left({Q}_h^{NT}\right)=1$, $U\left({Q}_h^T\right)=1-c$, $U\left({Q}_s^T\right)=b$, and $U\left({Q}_s^{NT}\right)=0$ without any loss of generality. Since the risk function *U* is increasing, 0 < *b* < 1 − *c* < 1. *b* can be interpreted as the utility of the therapeutic effect of the right treatment in the sick state. *c* can be viewed as the disutility of an adverse reaction to the wrong treatment in the healthy state. The agent is sick with probability *p* and healthy with probability 1 − *p*. Under the abovementioned setting, his/her regret-sensitive EU from treatment can be written as

$$ {W}_p^T=p\left[U\left({Q}_s^T\right)+ kg\left(U\left({Q}_s^T\right)-U\left({Q}_s^{NT}\right)\right)\right]+\left(1-p\right)\left[U\left({Q}_h^T\right)+ kg\left(U\left({Q}_h^T\right)-U\left({Q}_h^{NT}\right)\right)\right]=p\left[b+ kg(b)\right]+\left(1-p\right)\left[\left(1-c\right)+ kg\left(-c\right)\right]=p\left[b+c-1+ kg(b)- kg\left(-c\right)\right]+1-c+ kg\left(-c\right). $$

Likewise, his/her regret-sensitive EU from no treatment can be written as

$$ {W}_p^{NT}=p\left[U\left({Q}_s^{NT}\right)+ kg\left(U\left({Q}_s^{NT}\right)-U\left({Q}_s^T\right)\right)\right]+\left(1-p\right)\left[U\left({Q}_h^{NT}\right)+ kg\left(U\left({Q}_h^{NT}\right)-U\left({Q}_h^T\right)\right)\right]=p\left[0+ kg\left(-b\right)\right]+\left(1-p\right)\left[1+ kg(c)\right]=p\left[-1+ kg\left(-b\right)- kg(c)\right]+1+ kg(c). $$

Note that both types of regret-sensitive EU are linear functions of sickness probability *p*.

Regret (rejoicing) is denoted by a negative (positive) sign in the difference between utility under treatment and utility under no treatment.

For all *k* ∈ [0, ∞), it is easy to obtain^14^

$$ {W}_0^T<{W}_0^{NT},{W}_1^T>{W}_1^{NT}\ \mathrm{and}\ \frac{{\partial W}_p^T}{\partial p}>\frac{{\partial W}_p^{NT}}{\partial p} $$

**Fig. 1:**
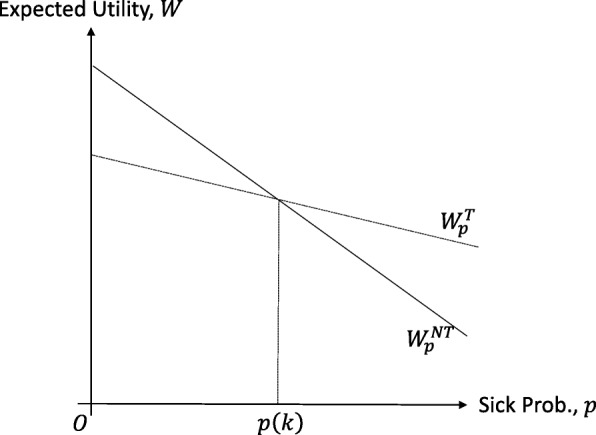
Sickness probability and regret-sensitive EU. This figure illustrates that the threshold probability, *p*(*k*), is the single crossing point between the regret-sensitive EU from treatment $\left({W}_p^T\right)$ and no treatment $\left({W}_p^{NT}\right)$, represented by the dotted and solid lines, respectively

These inequalities mean that ${W}_p^T$ and ${W}_p^{NT}$ satisfy the single-crossing property, that is, ${W}_p^T$ crosses ${W}_p^{NT}$ once from below at a single sickness probability. This is easily understood by viewing Fig. 1, in which the dotted and solid lines represent the regret-sensitive EU from treatment and no treatment, respectively. We denote this probability *p*(*k*) to explicitly indicate that it is dependent on regret sensitivity *k*, and call it the threshold probability. Probability *p*(*k*) provides the threshold probability by which the agent decides whether to undergo treatment or not because

$$ {W}_p^T>\left(=,<\right){W}_p^{NT}\iff p>\left(=,<\right)p(k). $$

In other words, the agent makes the treatment decision after comparing the sickness probability with the threshold probability. When the sickness probability is strictly higher (lower) than the threshold probability, the agent makes the decision to undergo (not to undergo) treatment. At threshold probability *p*(*k*), agent *k* is indifferent between treatment and no treatment. Threshold probability *p*(*k*) is given by

$$ p(k)=\frac{c+k\left(g(c)-g\left(-c\right)\right)}{b+c+k\left(g(b)-g\left(-b\right)\right)+k\left(g(c)-g\left(-c\right)\right)}. $$

## Results

Here, we examine how regret sensitivity *k* affects the threshold probability *p*(*k*).

The following theorem is our main result.

**Theorem 1.** *Suppose that the therapeutic effect in the sick state exceeds the adverse reaction in the healthy state, that is, b* ≥ *c. The following conditions are equivalent:*

- (*g*(*x*) − *g*(−*x*))/*x is increasing (decreasing) in x*
- *The threshold probability is decreasing (increasing) in the weight of regret sensitivity, that is,* $p\left(\underset{\_}{k}\right)\ge \left(\le \right)\ p\left(\overline{k}\right)$ *for all* $\underset{\_}{k},\overline{k}$ *with* $\underset{\_}{k}\le \overline{k}$ *.*

*Proof.* We denote *G*(*x*) = *g*(*x*) − *g*(−*x*) for notational simplicity. The sign of *dp*(*k*)/*dk* coincides with the sign of its numerator:

$$ G(c)\left(b+c+ kG(b)+ kG(c)\right)-\left(G(b)+G(c)\right)\left(c+ kG(c)\right)= bG(c)- cG(b). $$

Then,

$$ \frac{dp(k)}{dk}\le \left(\ge \right)0\iff bG(c)- cG(b)\le \left(\ge \right)0\iff \frac{g(b)-g\left(-b\right)}{b}\ge \left(\le \right)\frac{g(c)-g\left(-c\right)}{c}. $$

Since *b* ≥ *c*, the proof is complete.

Note that all of the variables in Theorem 1 (*b*, *c*, and *x*) are measured in utility units. It seems to be an innocuous assumption that the utility from the right treatment exceeds the disutility from the wrong treatment.^15^ This effect of regret sensitivity on the threshold probability is dependent on the shape of the regret–rejoicing function *g* in terms of how regret and rejoicing affect the threshold probability. Which condition, “(*g*(*x*) − *g*(−*x*))/*x* is increasing or is decreasing in *x*,” is reasonable? As shown in Bell [5], some choices that cannot be captured by EUT, such as the coexistence of insurance and gambling and probabilistic insurance (Kahneman and Tversky [12]), can be explained when the regret–rejoicing function satisfies the condition whereby (*g*(*x*) − *g*(−*x*))/*x* is increasing in *x*. Once this condition can be recognized from a descriptive viewpoint, introducing regret and rejoicing into the preference representation lowers the threshold probability. In other words, regret sensitivity causes the agent to tend to undergo treatment. In the remainder of this paper, we assume that (*g*(*x*) − *g*(−*x*))/*x* is increasing in *x*.

We provide an intuition of Theorem 1. When the agent undergoes treatment, there are two positions of merit in terms of regret and rejoicing: the agent enjoys rejoicing from making the right decision in the sick state, which is represented by *g*(*b*), and avoids regret from making the wrong decision in the sick state, which is represented by *g*(−*b*). A similar argument can be applied to the case in which the agent does not undergo treatment, that is, rejoicing is *g*(*c*) in the healthy state and regret is *g*(−*c*) in the healthy state. From the abovementioned argument, we can interpret Theorem 1 as follows. When the merit of treatment per utility unit ((*g*(*b*) − *g*(−*b*))/*b*) is greater than that of no treatment ((*g*(*c*) − *g*(−*c*))/*c*), the agent has a stronger incentive to undergo treatment, as the regret sensitivity *k* is larger.

As the EUT case corresponds to *k* = 0, we immediately obtain the following corollary.

**Corollary 1.** *Suppose that an agent has a regret-sensitive utility function where* (*g*(*x*) − *g*(−*x*))/*x is increasing in x. The threshold probability of the regret-sensitive agent is lower than that in the EUT case.*

In addition, we obtain the following intuitive results by simple calculations.

**Theorem 2.** *Assume that g is differentiable.*

- *The threshold probability is decreasing in the therapeutic effect of the treatment, that is, ∂p*(*k*)/*∂b* ≤ 0*.*
- *The threshold probability is increasing in the adverse reaction to the treatment, that is, ∂p*(*k*)/*∂c* ≥ 0*.*

**Fig. 2:**
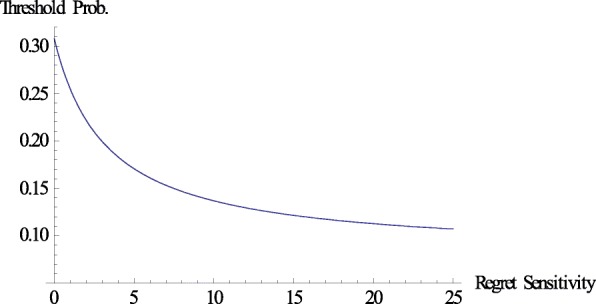
Regret sensitivity and threshold probability. This figure illustrates the relationship between regret sensitivity (*k*) and threshold probability. We can confirm the theoretical result presented in Theorem 1. In this numerical example, we set *g*(*x*) = *x*^3^*, b* = 0.45, and *c* = 0.2

**Fig. 3:**
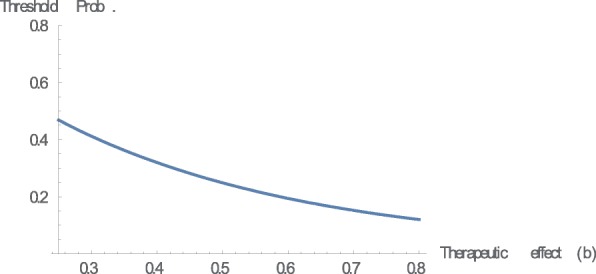
Therapeutic effect and threshold probability. This figure illustrates that the relationship between therapeutic effect (*b*) and threshold probability. We can confirm the theoretical result presented in Theorem 2. In this numerical example, we set *g*(*x*) = *x*^3^*, k* = 3, and *c* = 0.2

**Fig. 4:**
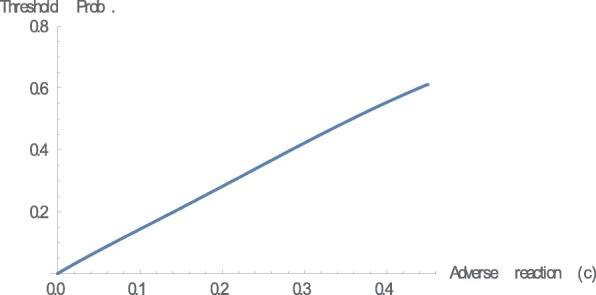
Adverse reaction and threshold probability. This figure illustrates that the relationship between adverse reaction (*c*) and threshold probability. We can confirm the theoretical result presented in Theorem 2. In this numerical example, we set *g*(*x*) = *x*^3^*, k* = 3, and *b* = 0.45

Finally, we provide a numerical example. Let us set *g*(*x*) = *x*^3^*, b* = 0.45, and *c* = 0.2. We can easily confirm that (*g*(*x*) − *g*(−*x*))/*x* is increasing in *x*. Figure 2 shows the relationship between regret sensitivity and the threshold probability. Let us consider the following simple example to obtain an intuitive understanding of the results based on the numerical example. An agent exhibits cold symptoms such as coughing and having a fever, which may also indicate the possibility of a severe illness such as pneumonia. Medical treatment is the right decision if the agent contracts a severe illness, and the agent gains the therapeutic effect, *b*. However, treatment is the wrong decision if he/she merely catches a slight cold, and the agent loses the adverse reaction, *c*. Suppose that the agent estimates the probability of a serious illness at 0.25. From Fig. 2, we conclude that the agent need not visit a doctor to undergo treatment when *k* is equal to zero, which is the EU case.^16^ However, the conclusion may be the opposite in regret theory. The agent should visit a doctor when, for example, *k* is equal to 3.^17^ It is important to classify whether the agent is sophisticated or naïve in terms of his/her regret sensitivity. When the agent is naïve, he/she cannot recognize that the medical decision is dependent on his/her regret sensitivity. We also examine the effect of the therapeutic effect of the treatment (*b*) and the adverse reaction to the treatment (*c*) on the threshold probability based on the above numerical example, where we set *k* = 3 (Figs. 3 and 4).

## Discussion

Many OECD countries provide public medical services through universal health-care programs (UHCPs). The United States was previously an exception until a UHCP (known as Obamacare) was introduced in 2010.^18^ In this section, we demonstrate how regret sensitivity may cause problems in UHCPs and suggest a solution to help mitigate these problems. We discuss two problems: (i) regret sensitivity leads to higher medical costs than the government has forecast and (ii) regret sensitivity causes inequality of benefits from public medical services.

Before proceeding with the exposition, we introduce the notion of willingness to pay (WTP) for treatment, which is defined as the maximum amount an agent can pay to undergo treatment. The threshold probability *p*(*k*) can be transformed into a monetary value by *WTP*(*k*), which represents the WTP of agent *k*. From Theorem 1, we show that the WTP of a regret-sensitive agent $\overline{k}$ is higher than that of a less regret-sensitive agent $\underset{\_}{k}\ \left(\le \overline{k}\right)$*, that is,* $WTP\left(\overline{k}\right)\ge WTP\left(\underset{\_}{k}\right)$.^19^

Under UHCPs, an agent only pays a part of the actual cost of treatment, with the remainder being paid for by subsidies collected from the entire nation through taxes and/or insurance premiums. We call the amount of agents’ own payments their “medical expenses,” and denote this by *ME*.^20^

### Increases in medical expenses and inequality of benefits

Here, we show that regret sensitivity may increase medical cost the government bears than one the government forecasts. Let us consider agents to be homogeneous, apart from their sickness probabilities, which are uniformly distributed over [0, 1] by normalization. A policymaker determines the subsidy necessary to maximize social welfare and estimates that the optimal proportion of the population undergoing treatment is 1 − *p*^∗^. EUT has traditionally been the dominant tool, and is considered to be a suitable normative criterion in medical decision-making. Based on EUT, the subsidy should be set at *p*^∗^ = *p*(0). Recall that *k* = 0 corresponds to EUT. Here, we consider that agents maximize their regret-sensitive EU in reality, even though EUT is the basis of policymaking. The strength of regret sensitivity is denoted by *k* > 0. Given the medical expenses based on EUT, the threshold probability becomes *p*(*k*), which is less than *p*(0). Thus, 1 − *p*(*k*) of the population undergo treatment. As a result, the government will incur higher medical costs than expected, that is, *p*(0) − *p*(*k*) times the subsidy. The above argument suggests that medical cost the government bear may become too high if regret sensitivity is taken into consideration in medical decision-making.

Regret sensitivity might also lead to inequality of benefits from medical services in UHCPs. Let us assume that agents have different strengths of regret sensitivity *k* ∈ [0, 1], and are identical in all other aspects. We denote the WTP for the treatment of agent *k* as *WTP*(*k*). The medical expense, *ME*, is set as *WTP*(0) ≤ *ME* ≤ *WTP* (1). There exists *k*^*O*^ such that *ME* = *WTP* (*k*^*O*^). If regret sensitivity *k* is more (less) than *k*^*O*^, agent *k* tends to undergo (not to undergo) treatment because *ME* = *WTP*(*k*^*O*^) ≤ (≥)*WTP*(*k*) for *k*^*O*^ ≤ (≥)*k*. This means that agents whose regret sensitivity is greater than *k*^*O*^ can benefit from all of the medical services provided by UHCPs, even though the subsidy is collected from the entire nation, including agents with lower regret sensitivity, that is, *k* < *k*^*O*^. This inequality causes the only difference in regret sensitivity in our setting. From this observation, UHCPs need some mechanisms by which they can guarantee equality, regardless of the differences in regret sensitivity.

### Suggested solution

Here, we suggest a solution to the problems caused by regret sensitivity. In this subsection, we consider the example used in the Results section. Let us consider cold symptoms, which are also related to the probability of a severe illness such as pneumonia. The probability of severe illness is denoted as *p*. If the agent visits a doctor, he/she gains the benefit (*b*) of receiving treatment when the illness is severe, but incurs a cost in terms of money and time (*c*).^21^

Let us consider two agents, $\overline{k}$ and $\underset{\_}{k}$, with $\overline{k}>\underset{\_}{k}$. We assume the following situation. Both agents display cold symptoms twice a year, and the medical expense lies at $WTP\left(\overline{k}\right)> ME> WTP\left(\underset{\_}{k}\right)$. In this situation, agent $\overline{k}$ visits a doctor twice a year, but agent $\underset{\_}{k}$ never visits a doctor. As a result, agent $\overline{k}$ can obtain a benefit from UHCPs, but agent $\underset{\_}{k}$ cannot. If the medical expense is set such that it increases with the times to visit a doctor, it may be possible to prevent this inequality caused by regret sensitivity. For example, consider the case in which the medical expense is set to $ME(2)> WTP\left(\overline{k}\right)> WTP\left(\underset{\_}{k}\right)> ME\ (1)$, where *ME* (*i*) denotes the medical expense of *i* (*i* = 1, 2) visits to a doctor. In this setting, both agents visit a doctor once a year, so there is no inequality between patient $\overline{k}$ and patient $\underset{\_}{k}$. This might also reduce medical costs because it prevents highly regret-sensitive agents from visiting a doctor numerous times.

The early detection of serious illness is important. Doctors may find signs of serious illness such as pharyngeal cancer when agents present with what appear to be merely cold symptoms. The possibility of early detection diminishes per examination. Thus, equality of access to medical treatment is important not only for reducing medical costs, but also for the early detection of serious illness in more people.

In this section, we consider the situation in which the decision-maker is an agent. However, a similar argument can also be applied to other situations, for example, where the medical decision is made by the agent’s general practitioner (GP). The GP decides whether to refer an agent to a specialist based on their estimation of the likelihood of severe illness following a medical examination of what initially appear to be cold symptoms. When the GP is naïve in relation to regret sensitivity in medical decision-making, some variations to our suggested solution might be promising ways to mitigate the problems. On one hand, the GP is somewhat reluctant to refer an agent to a specialist if he/she does not appear to have a serious illness. On the other hand, the GP is fearful of missing a severe illness. In this situation, the GP might be reluctant to routinely refer the agent to a specialist if the GP is unsure that the agent has a severe illness. This kind of pressure is harmful to a society based on EUT. However, this kind of pressure may be useful in mitigating the problems caused by the presence of regret sensitivity.

Because the example considered in this section is a simplification of the complexity in the real world, we recognize that our suggested solution cannot be directly applied to solving real-world problems. However, this suggested solution might be a useful as a starting point for a discussion on policy intervention in relation to medical treatment to mitigate social problems.

## Conclusion

In this study, regret and rejoicing are incorporated into the classical medical decision-making problem presented by Pauker and Kassirer [1, 2]. When the regret–rejoicing function satisfies the condition that (*g*(*x*) − *g*(−*x*))/*x* is increasing in *x*, the threshold probability decreases as a result of regret sensitivity. In other words, agents with higher regret sensitivity tend to opt for more medical treatment.

In a recent study, Felder and Mayrhofer [9] showed that the threshold probability decreases when risk vulunable agents face comorbidity risk in addition to diagnostic risk. This might explain empirical findings of lower threshold probabilities than those predicted by the classical model of Pauker and Kassirer [1, 2]. It is interesting that we obtain the same result as Felder and Mayrhofer [9], even though our approaches differ.

The limitations of this study are its simple setting, namely, a binary state and a binary choice. While this setting is necessary for the treatment threshold approach, we should relax these assumptions in future research. The binary choice is essential because the original regret theory, which is adopted in this study, causes intransitivity in choices from among more than two alternatives. To avoid this difficulty, we need to adopt modified versions of regret theory satisfying transitivity, for example, the preference representation proposed by Braun and Muermann [10].

It goes without saying that we need a parametric form of regret-sensitive utility function for actual applications. Even though measurement methods to elicit preference parameters of regret theory have been developed, for example by Bleichrodt et al. [11], we need to collect more experimental and empirical evidence to obtain consensus regarding plausible parametric forms of the regret-sensitive utility function. In addition to addressing the limitations of this study, this is an important future line of research regarding the application of regret theory. Qualitative analysis, including that presented in this study, will also play a complementary role in examining the descriptive properties of specific forms of the regret-sensitive utility function.

## Appendix

In introducing WTP, we consider a bivariate risk function whose first argument is monetary wealth and second argument is QALYs, *U*(*M*, *Q*). The bivariate risk function is assumed to be increasing in both the first and second arguments, that is, *U*_1_, *U*_2_ > 0. WTP is defined as the maximum monetary amount agents are willing to pay for treatment. Agent *k* is endowed with initial wealth *M*. *WTP*(*k*) is referred to as the WTP of agent *k*. Final wealth can be written as *M*^*T*^(*k*) = *M* − *WTP*(*k*) when the agent undergoes treatment and *M*^*NT*^ = *M* when the agent does not undergo treatment. As noted in the main text, utility is set as $U\left({M}^{NT},{Q}_h^{NT}\right)=1$, $U\left({M}^T,{Q}_h^T\right)=1-c(k)$, $U\left({M}^T,{Q}_s^T\right)=b(k)$, and $U\left({M}^{NT}{Q}_s^{NT}\right)=0$. We retain the assumption that 0 < *b*(*k*) < 1 − *c*(*k*) < 1. WTP satisfies the following condition:

$$ {\displaystyle \begin{array}{c}{W}_p^T\left(k, WTP(k)\right)=p\left[b(k)+ kg\left(b(k)\right)\right]+\left(1-p\right)\left[\left(1-c(k)\right)+ kg\left(-c(k)\right)\right]\\ {}=p\left[ kg\left(-b(k)\right)\right]+\left(1-p\right)\left[1+ kg\left(c(k)\right)\right]={W}_p^{NT}\left(k, WTP(k)\right).\end{array}} $$

Given the WTP, the above condition is identical to the threshold probability.

From Theorem 1, we obtain the following inequality:

$$ {W}_p^T\left(\overline{k}, WTP(k)\right)\ge {W}_p^{NT}\left(\overline{k}, WTP(k)\right) $$

for all $\overline{k}$ where $\overline{k}\ge k$. This means that agent $\overline{k}$ can pay additional costs to undergo treatment. As a result, we obtain $WTP\left(\overline{k}\right)\ge WTP(k)$. We set $k=\underset{\_}{k}$, and $WTP\left(\overline{k}\right)\ge WTP\left(\underset{\_}{k}\right)$ holds for $\overline{k}$ and $\underset{\_}{k}$ with $\overline{k}\ge \underset{\_}{k}$.

## Footnotes

1 Comprehensive surveys of medical decision-making were undertaken by Eeckhoudt [12] and Felder and Mayrhofer [13]. Djulbegovic et al. [14] present a review of recent developments in the treatment threshold approach.

2 Although we adopt “regret theory” in accordance with this custom, the name may be misleading because we must keep in mind that rejoicing is also included in regret theory.

3 We have borrowed the word “bold” from Bleichrodt and Wakker [15].

4 Bleichrodt and Wakker [15] provide an excellent survey of the development of regret theory.

5 A series of studies and other related literature are included in the references therein.

6 In this approach, ranges of sickness probabilities are determined, in which people feel regret below specified levels by undergoing medical treatment.

7 As noted in Hozo and Djulbegovic [8] (p. 542), “the complexity of modelling negative regret values and nonlinear value functions in a medical setting makes this original economic definition of regret difficult to apply.”

8 Since the threshold probabilities based on both EUT and the linear regret function coincide, earlier studies needed to adopt a different approach such as acceptable regret.

9 The regret utility function is cardinal in the sense that it is unique up to the affine transformation.

10 Because we do not consider any optimization problems in our analysis, no regularity conditions for optimality are necessary.

11 The regret-sensitive utility function also corresponds to EUT when the regret–rejoicing function is linear.

12 In general, treatment decisions are not necessarily made by the agent. Decisions might be taken by his/her family, his/her general practitioner (GP), or someone else.

13 The health outcome is measured in QALYs for ease of exposition. We can adapt any other variables if the health outcome is measurable and unidimensional.

14 may be positive when regret sensitivity is sufficiently large.

15 If is less than, we obtain the same statement by reversing either of the conditions.

16 For ease of exposition, we consider the situation in which an agent decides whether to visit a specialist or not. The same explanation can be applied to other situations by changing roles. For example, an agent goes to see a GP, and the GP makes the decision to refer the agent to a specialist or not.

17 The threshold probability is 0.25 when is about 1.12.

18 The Obamacare was reviewed under Trump administration.

19 A formal argument is presented in the Appendix.

20 The actual costs are equal to the sum of the medical expenses and subsidies.

21 Money and time are evaluated in terms of QALYs.
